# Supplementary material for: Explorations on Thermodynamic and Kinetic Performances of Various Cationic Exchange Durations for Synthetic Clinoptilolite
Source: Molecules. 2022 Apr 18;27(8):2597. doi: 10.3390/molecules27082597 (PMC9024986; doi:10.3390/molecules27082597)
Supplement: Supplementary file 1 [file molecules-27-02597-s001.zip › molecules-1653227-supplementary.pdf]

Explorations on thermodynamic and kinetic performances of various cationic exchange durations for synthetic clinoptilolite

Keling Wang, Bingying Jia, Yehong Li, Jihong Sun*, Xia Wu*

Beijing Key Laboratory for Green Catalysis and Separation, Department of Environmental and Chemical Engineering, Beijing University of Technology, Beijing, 100124, China

Electronic Supplementary Information

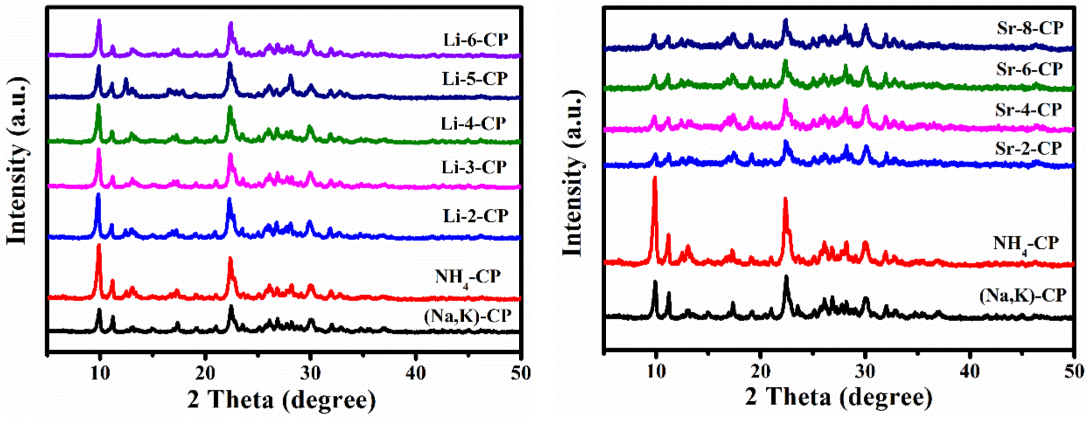

(A) (B)

Figure S1. XRD patterns of Li-x-CP (A) and Sr-x-CP (B).

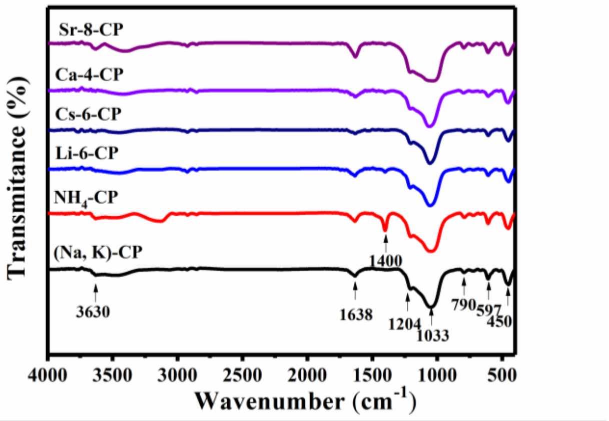

Figure S2. FT-IR spectra of various CPs.

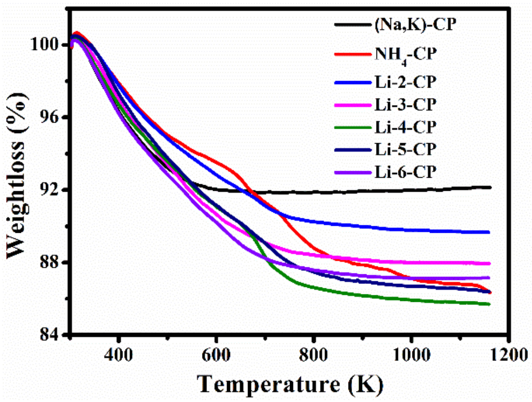

(A)

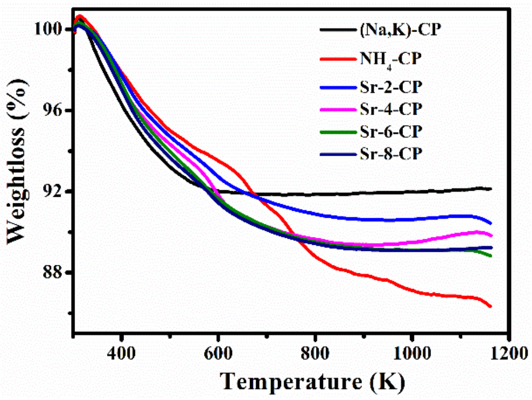

(B)

Figure S3. TG profiles of Li-x-CP (A) and Sr-x-CP (B).

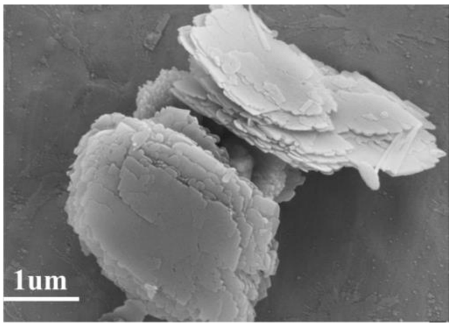

(A)

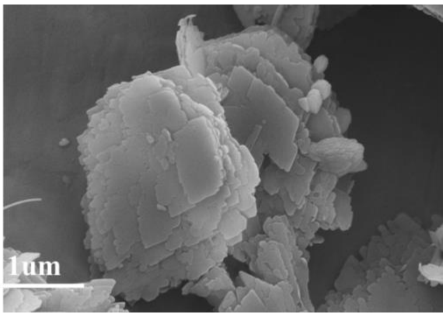

(B)

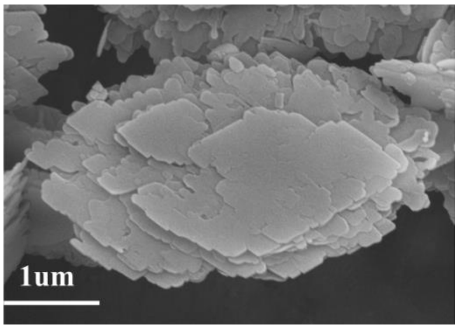

(C)

Figure S4. SEM images of (Na, K)-CP (A), Cs-6-CP (B), and Ca-4-CP (C).

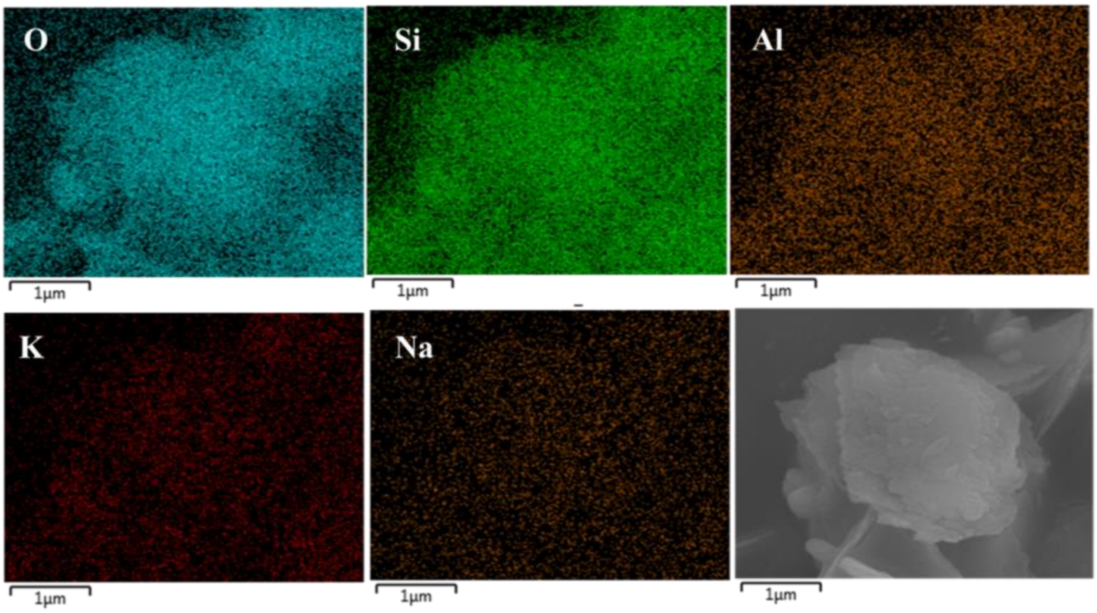

Figure S5. SEM elemental mappings of (Na, K)-CP.

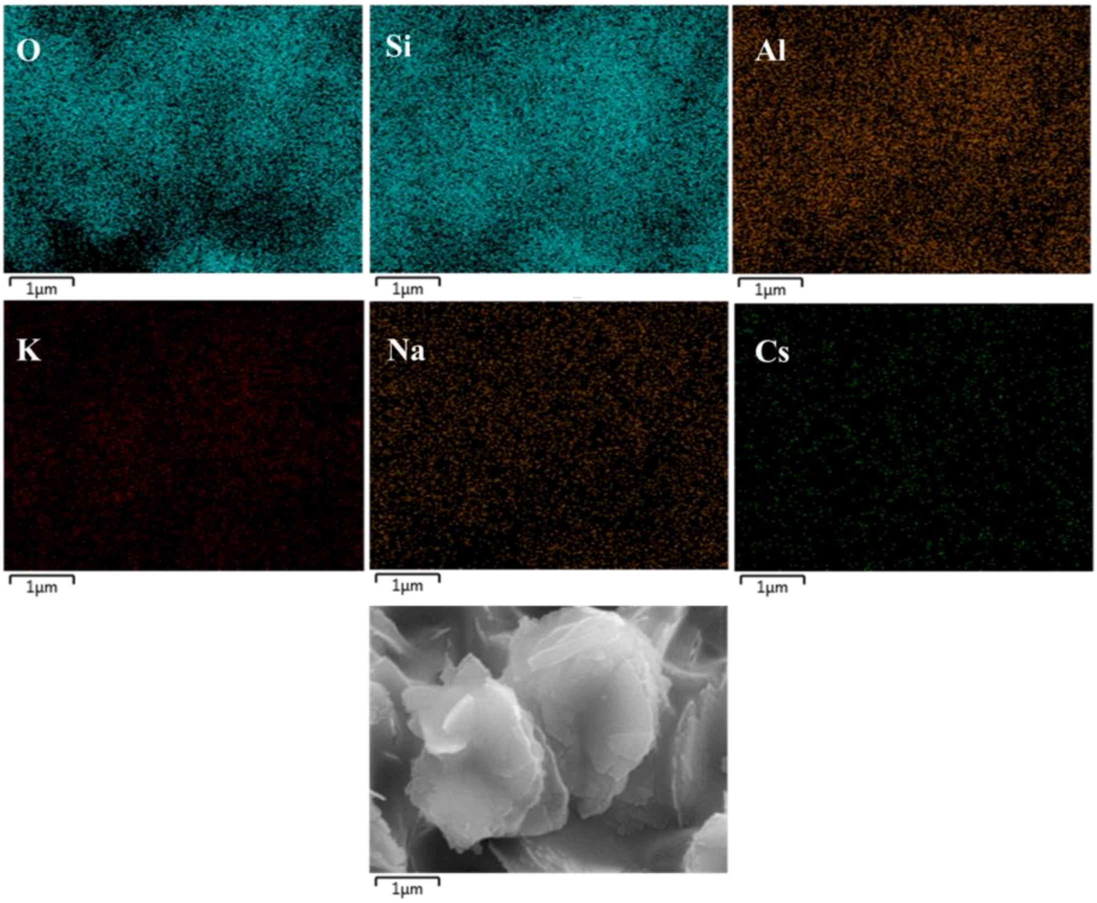

Figure S6. SEM elemental mappings of Cs-6-CP.

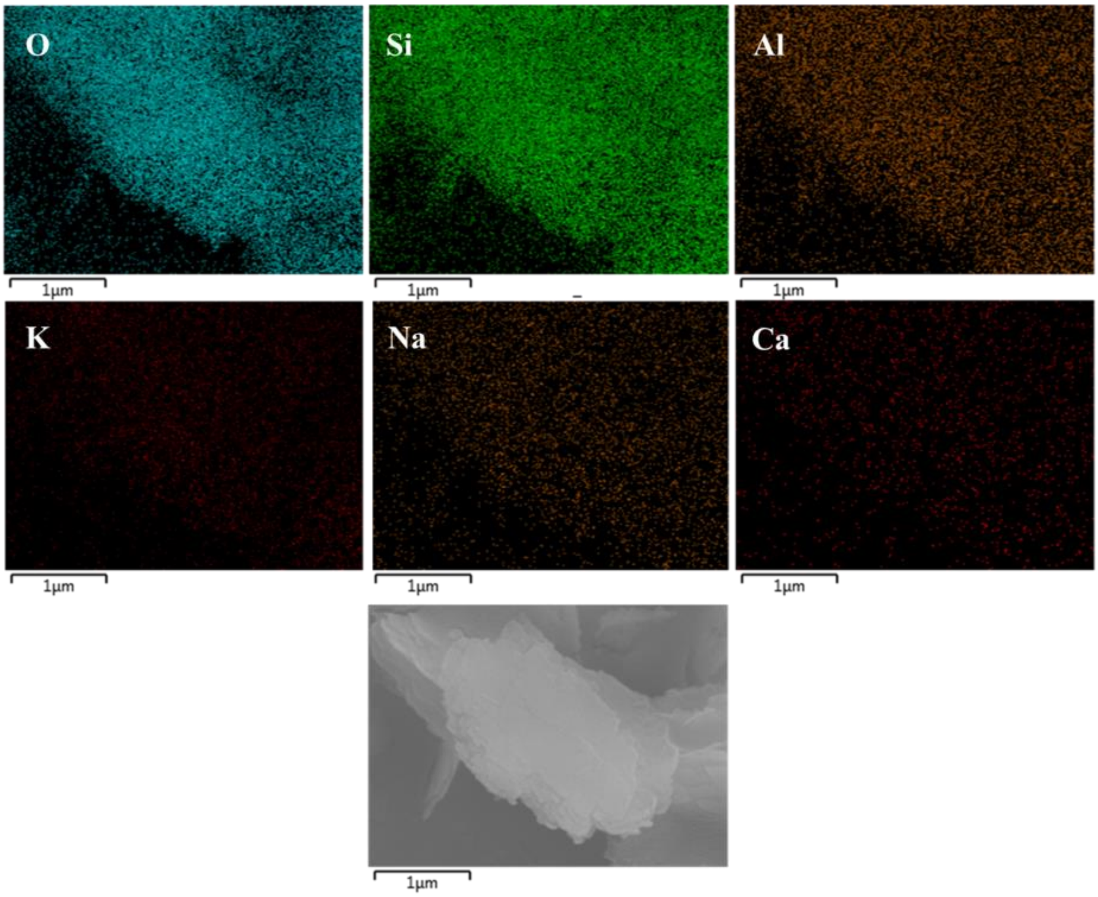

Figure S7. SEM elemental mappings of Ca-4-CP.

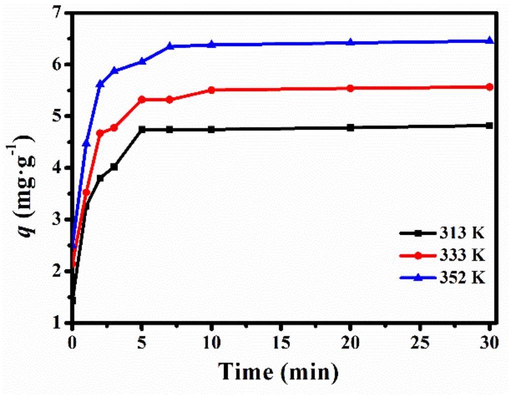

(A)

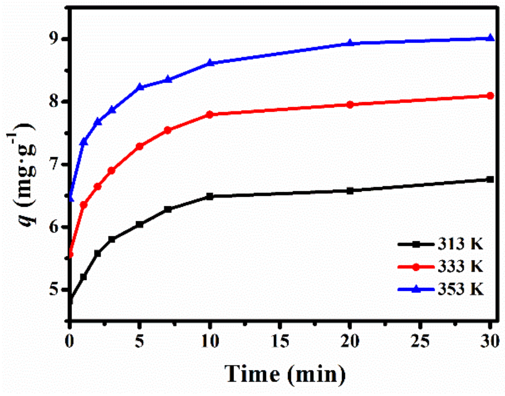

(B)

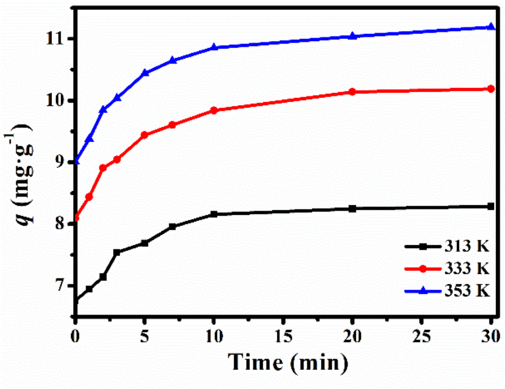

(C)

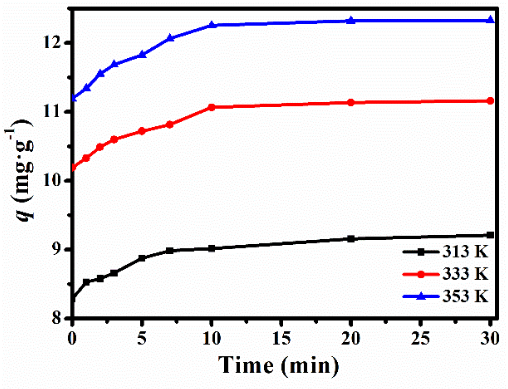

(D)

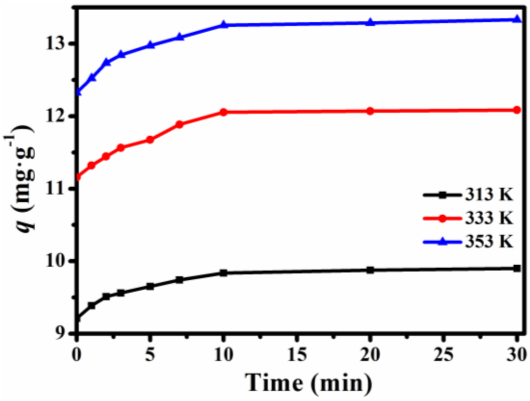

(E)

Figure S8. Exchanged kinetic curves of Li-x-CP at 313 K, 333 K, and 353 K. x = 2 (A), 3 (B), 4 (C), 5 (D), and 6 (E).

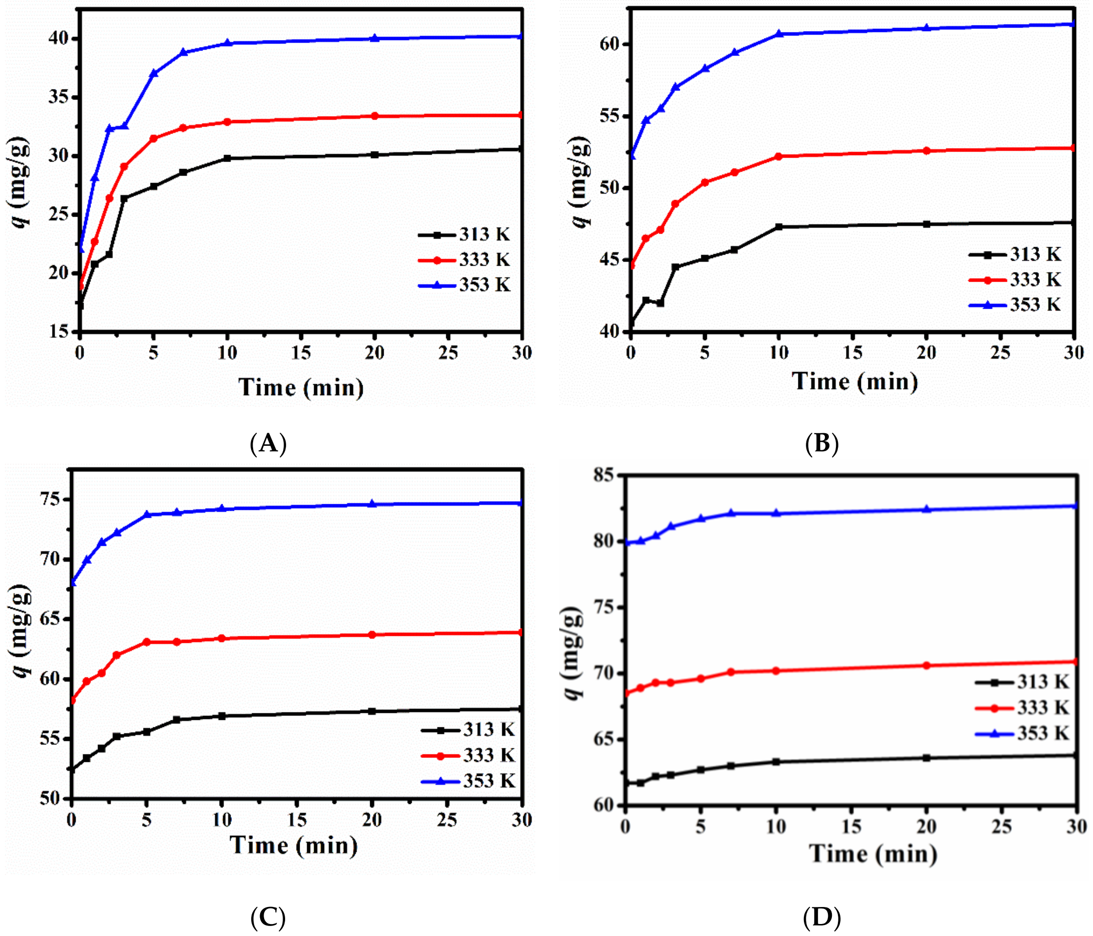

Figure S9. Exchanged kinetic curves of Sr-x-CP at 313 K, 333 K, and 353 K. x = 2 (A), 4 (B), 6 (C), and 8 (D).

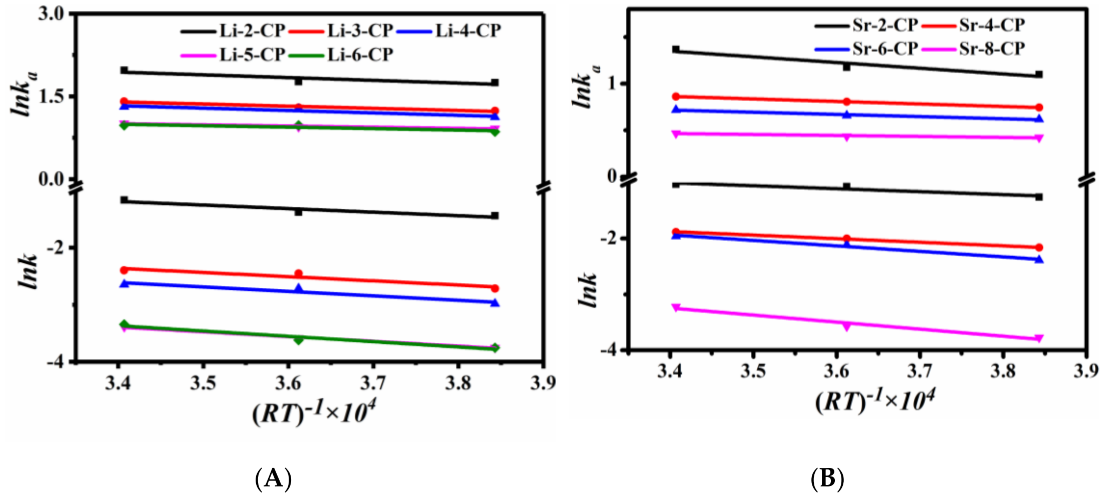

Figure S10. Relationship between $ln\ k_a$ and $(RT)^{-1}\times10^4$, as well as $ln\ k$ and $(RT)^{-1}\times10^4$ for Li-x-CP (A) and Sr- x-CP (B).

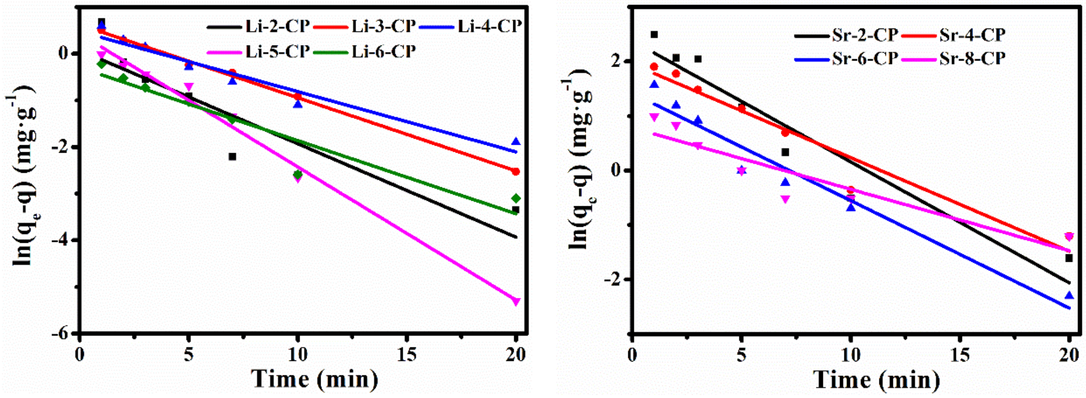

(**A**) (**B**)

Figure S11. Pseudo-first-order model fit for Li-x-CP (A) and Sr-x-CP (B).

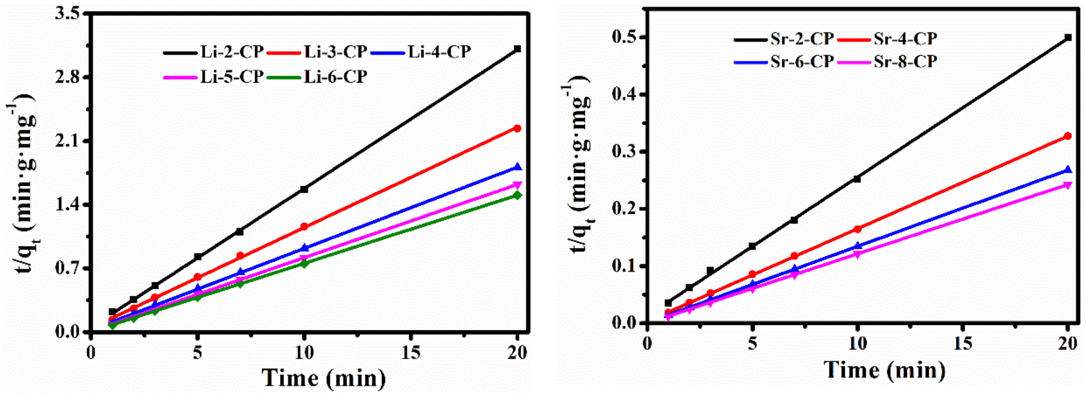

(**A**) (**B**)

Figure S12. Pseudo-second-order model fit for Li-x-CP (A) and Sr-x-CP (B).

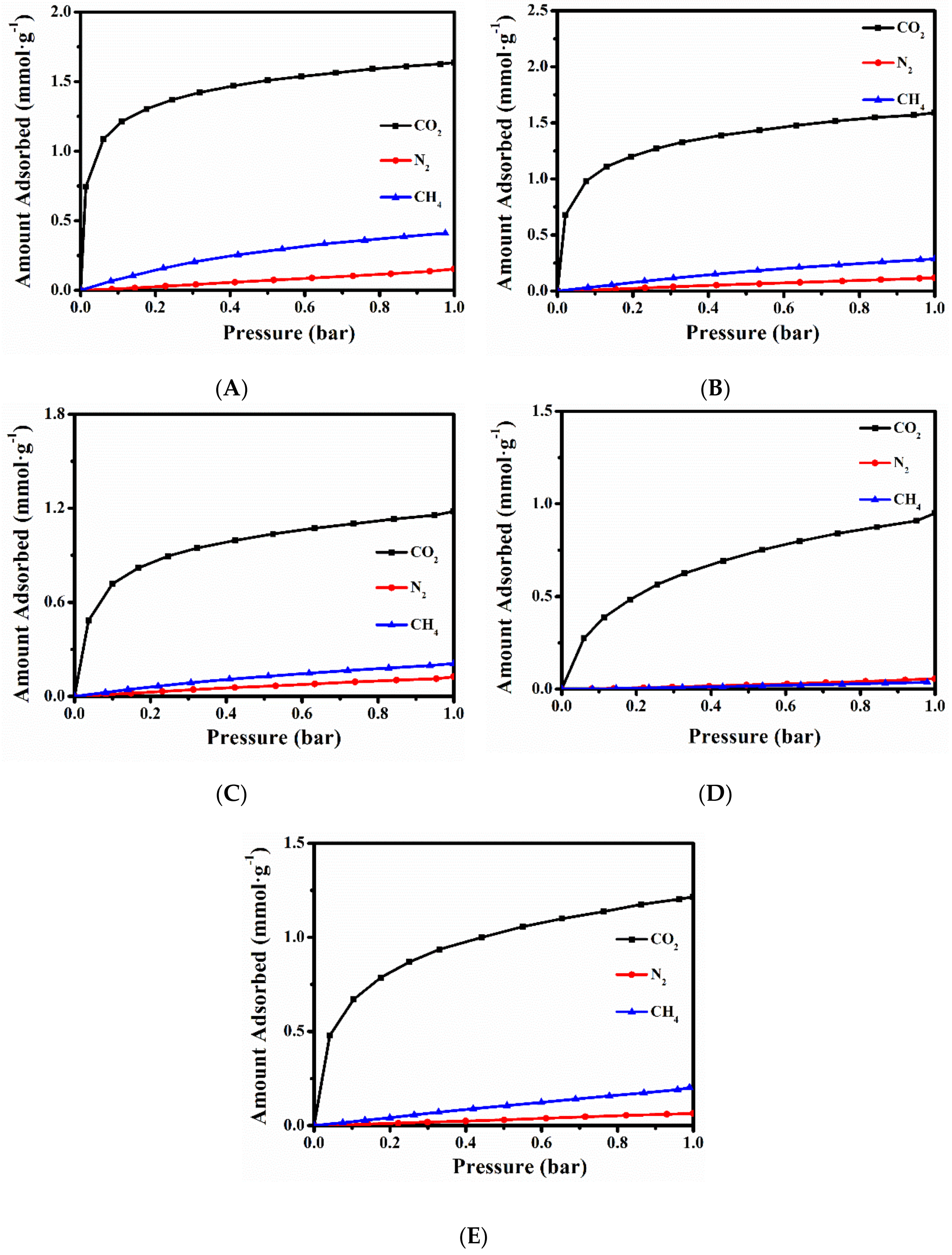

Figure S13. Adsorbed isotherms of (Na, K)-CP (A), Li-6-CP (B), Cs-6-CP (C), Ca-4-CP (D), and Sr-8-CP (E) using CO_2, N_2 and CH_4 as adsorbate at 298 K.

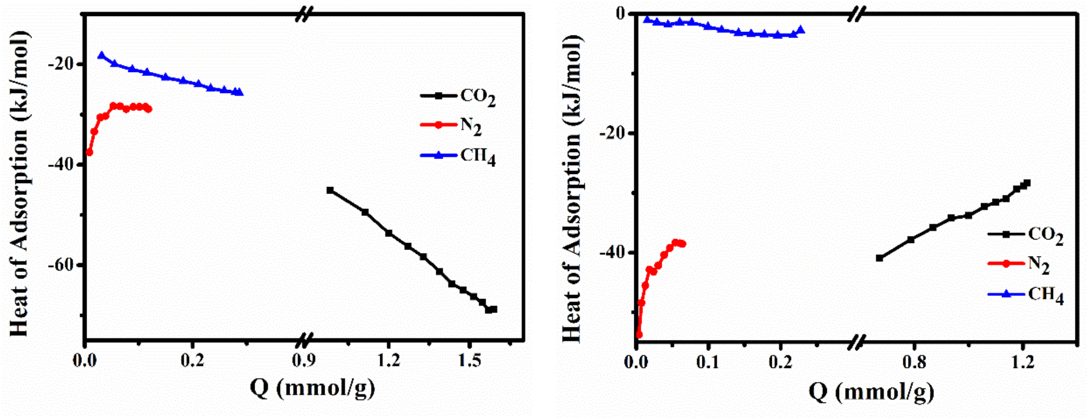

(A) (B)

Figure S14. Adsorption heat of CO_2, N_2 and CH_4 of Li-6-CP (A) and Sr-8-CP (B).

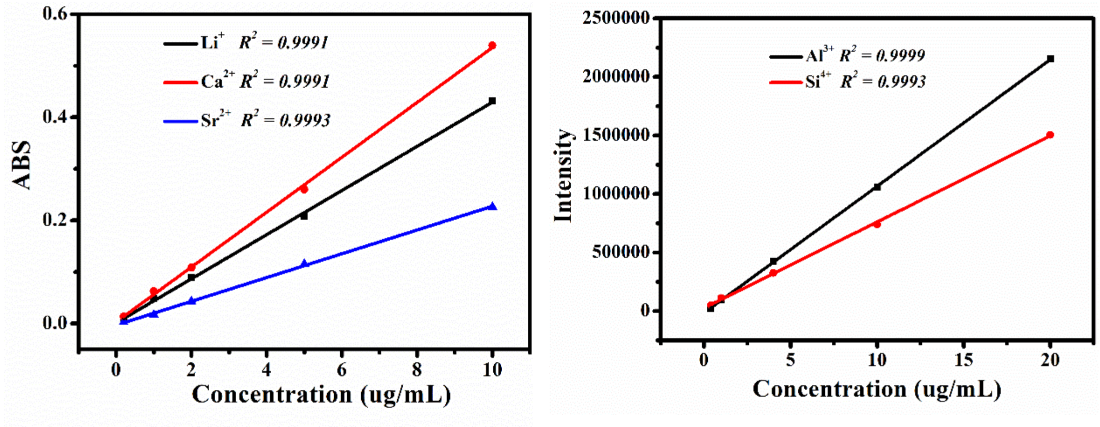

(A) (B)

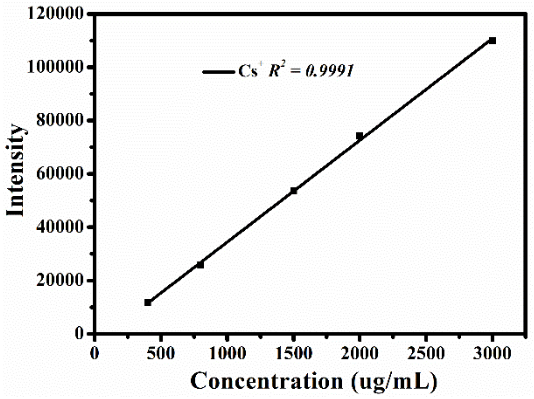

(C)

Figure S15. The standard curves of Li^+, Ca^{2+}, Sr^{2+} (A), Al^{3+}, Si^{4+} (B), and Cs^+ (C).

Table S1. Summaries for the *d* (nm) spacing values of the obtained M-x-CPs corresponding to different spatial planes.

Samples	*020*	*200*	*111*	*13-1*	*131*	*22-2*	*42-2*	*151*	*530*	*061*
CP	8.909	7.892	5.104	4.637	3.959	3.402	3.160	2.970	2.796	2.725
NH_4-CP	8.927	7.909	5.132	4.648	3.976	3.414	3.162	2.980	2.800	2.734
Li-2-CP	8.982	7.963	5.133	4.666	3.976	3.427	3.173	2.988	2.802	2.728
Li-3-CP	8.946	7.950	5.139	4.651	3.979	3.416	3.175	2.965	2.801	2.730
Li-4-CP	8.927	7.907	5.109	4.633	3.963	3.411	3.166	2.974	2.799	2.730
Li-5-CP	8.984	7.953	5.145	4.649	3.983	3.429	3.175	2.988	2.807	2.730
Li-6-CP	8.962	7.907	5.105	4.642	3.973	3.414	3.166	2.984	2.803	2.735
Cs-2-CP	9.055	7.996	5.169		4.005	3.432	3.173	2.996	2.817	2.743
Cs-3-CP		8.025			4.005	3.432	3.177	3.002	2.819	2.746
Cs-4-CP	9.020	8.065			4.004	3.438	3.177	3.013		2.746
Cs-5-CP	9.157	8.007	5.164	4.709	4.001	3.435	3.179	2.998		2.751
Cs-6-CP		8.025			4.005	3.432	3.177	3.002	2.819	2.746
Ca-2-CP	8.983	7.937	5.121	4.661	3.973	3.424	3.182	2.961	2.797	2.730
Ca-3-CP	8.965	7.922	5.115	4.649	3.973	3.422	3.173	2.963	2.796	2.726
Ca-4-CP	8.944	7.906	5.082	4.647	3.952	3.411	3.169	2.961	2.787	2.735
Sr-2-CP	8.876	7.866	5.087	4.628	3.952	3.412	3.160	2.959	2.791	2.720
Sr-4-CP	8.965	7.921	5.099	4.643	3.973	3.421	3.171	2.968	2.798	2.730
Sr-6-CP	8.990	7.948	5.122	4.657	3.976	3.424	3.173	2.965	2.801	2.733
Sr-8-CP	9.035	7.908	5.105	4.643	3.969	3.417	3.175	2.972	2.800	2.734
